# Editorial: Advances in Inorganic Materials for Supercapacitors and Batteries

**DOI:** 10.3389/fchem.2022.955942

**Published:** 2022-06-15

**Authors:** Keyvan Malaie, Yohan Dall’Agnese, Sayed Habib Kazemi

**Affiliations:** ^1^ Institute of Biochemistry, University of Greifswald, Greifswald, Germany; ^2^ Institute for Materials Discovery, University College London, London, United Kingdom; ^3^ Department of Chemistry, Institute for Advanced Studies in Basic Sciences (IASBS), Zanjan, Iran

**Keywords:** batteries, supercapacitors, inorganic materials, metal oxides, ion insertion

**FIGURE 1:**
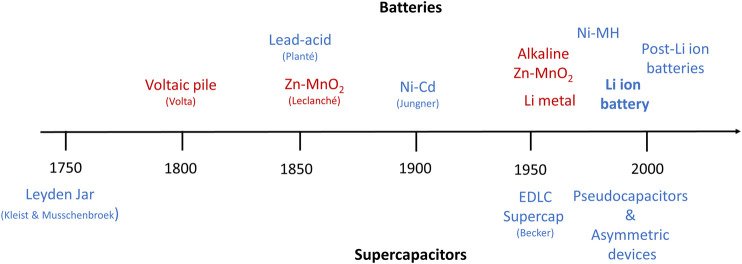
A brief history of batteries and supercapacitors (red: primary, blue: rechargeable).

Batteries and supercapacitors have become important elements of everyday life, thanks to the extensive effort contributed to their development. It is interesting to look back at the historical development of batteries and supercapacitors during the last two centuries since the invention of the first battery—called voltaic pile—by Alessandro Volta in 1800 and the first patent on electrochemical double layer capacitors by H. I. Becker in 1957 (Figure 1). A survey of this timeline reveals that 1) progress happens rather incrementally over a long time with few paradigm changes such as lithium ion batteries, and 2) while early developments may be attributed to specific individuals, recent advances are the result of extensive collaborative work of many researchers from different disciplines. Indeed, such schematic illustrations only list the outcome technologies, and the implicit influence of many material scientists, electrochemists, and physicists who discovered new materials, developed double layer theories, and introduced new concepts such as ion insertion/deinsertion cannot be overlooked.

It often happens that improving the performance of an energy storage system becomes increasingly difficult as the system approaches the practically-attainable power/energy density which is usually 50%–60% of the theoretical limit. This along with the insufficient cycling stability especially in the case of batteries constitute two of the most important challenges in today’s energy storage research. Therefore, a marked further development of batteries and supercapacitors definitely requires both fundamental and applied research covering various aspects such as the underlying electrochemical/electronic processes, structure-performance relationships, new storage mechanisms, as well as material discovery and optimization. Accordingly, in the last 2 decades several new research directions have been followed that may be all together called “post-lithium ion batteries.” The most important ones are probably solid state batteries, flow batteries, lithium metal batteries (Li/O_2_ and Li/S), as well as sodium ion and dual-ion insertion batteries.

The present Research Topic aims at reflecting on the recent advances in batteries and supercapacitors based on inorganic materials. The electrode materials based on inorganics possess tunable properties such as various metal oxidation states, diverse crystal phases, high amenability to defects/vacancies, synergetic intermetallic effects, and so on; hence, there is a large playground for improving their electrochemical performances. A good example of how introducing vacancy (or defect) through metal ion doping can significantly influence the electrochemical performance is presented here by Le Calvez et al. They have doped the perovskite structure AgNbO_3_ partially by La^3+^ and observed a substantial enhancement on its capacity for lithium ion insertion/deinsertion. Interestingly, the defective perovskite Ag_1-3x_La_x_NbO_3_ shows no detectable phase transition during lithium insertion/deinsertion and remains a solid solution. Akintola et al., on the other hand, have modified an anionic MOF with lithium and sodium ions through ionic exchange and investigated them as anodes for lithium and sodium ion batteries. The as-treated MOF electrode exhibits an improved capacity and stability. Norouzi et al. report on the preparation of an electrode based on MoO_3_/carbon by a sol-gel/hydrothermal process and show that the capacitance of the electrode in aqueous ZnCl_2_ electrolyte improves due to a carbonization process. Finally, Esteve-Adell et al. have studied the lithium ion storage capacity of graphene nanoplatelets with different surface area as anodes for lithium-ion batteries and have concluded that the introduction of defect/disorder into the graphene structure through porosification is important for enhancing capacity.

Last but not least, as Research Topic Editors, we would like to thank all authors, peer-reviewers, and the Frontiers’ team for their valuable contributions to this Research Topic, and hope the readers will appreciate the reported findings as much as we did.

